# Nonhost species reduce parasite infection in a focal host species within experimental fish communities

**DOI:** 10.1002/ece3.7823

**Published:** 2021-06-29

**Authors:** Sangwook Ahn, Cameron P. Goater

**Affiliations:** ^1^ Department of Ecosystem and Public Health, Faculty of Veterinary Medicine University of Calgary Calgary AB Canada; ^2^ Department of Biological Sciences University of Lethbridge Lethbridge AB Canada

**Keywords:** cercariae, dilution effect hypothesis, introduced fish, parasite ecology, trematode, wildlife epidemiology

## Abstract

The dilution effect describes the negative association between host biodiversity and the risk of infectious disease. Tests designed to understand the relative roles of host species richness, host species identity, and rates of exposure within experimental host communities would help resolve ongoing contention regarding the importance and generality of dilution effects. We exposed fathead minnows to infective larvae of the trematode, *Ornithodiplostomum ptychocheilus* in minnow‐only containers and in mixed containers that held 1–3 other species of fish. Parasite infection was estimated as the number of encysted worms (i.e., brainworms) present in minnows following exposure. The results of exposure trials showed that nonminnow fish species were incompatible with *O. ptychocheilus* larvae. There was no reduction in mean brainworm counts in minnows in mixed containers with brook sticklebacks or longnose dace. In contrast, brainworm counts in minnows declined by 51% and 27% in mesocosms and aquaria, respectively, when they co‐occurred with emerald shiners. Dilution within minnow + shiner containers may arise from shiner‐induced alterations in minnow or parasite behaviors that reduced encounter rates between minnows and parasite larvae. Alternatively, shiners may act as parasite sinks for parasite larvae. These results highlight the role of host species identity in the dilution effect. Our results also emphasize the complex and idiosyncratic effects of host community composition on rates of parasite infection within contemporary host communities that contain combinations of introduced and native species.

## Graphical abstract

The effects of host diversity on rates of parasite infection are context‐dependent. When fathead minnows swim in containers with one non‐host competitor (longnose dace), rates of infection into minnows are the same as when minnows swim in containers by themselves. In contrast, when minnows swim in containers with another species of non‐host competitor (emerald shiners), infection rates decrease by more than 50%.

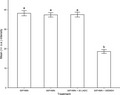

## 1 INTRODUCTION

Research concerning the biodiversity–disease relationship over the past 20 years has emphasized how changes in the diversity of hosts in a community, many of which are anthropogenically induced, influence the risk of disease transmission into a focal species of host (Halliday & Rohr, 2019; Johnson & Thieltges, 2010). Tests of the dilution effect hypothesis, which suggests that increased biodiversity (of both host and nonhost species) reduces infection risk within host communities, have been central to this research focus (Civitello et al., 2015; Keesing et al., 2006). The results of such tests have shown that increases in biodiversity can dilute the risk of transmission of infective stages of important human (e.g., Lyme disease—Ostfeld & Keesing, 2000; West Nile virus—Allan et al., 2009; hantavirus—Suzán et al., 2009) and wildlife (Johnson et al., 2013) parasites. Empirical evidence consistent with a dilution effect is present in numerous host–parasite and plant–herbivore systems (Civitello et al., 2015). However, there is accumulating evidence that the direction and magnitude of biodiversity–disease relationships can be highly context‐dependent, leading to idiosyncratic outcomes in which factors such as relative host species density, parasite transmission strategy, parasite specificity, host identity, and relative host compatibility play a key role (Halliday & Rohr, 2019). Such complexity has called into question the generality of the dilution effect and has increased the call for mechanistic tests designed to determine the ecological conditions when dilution effects are most likely to occur (Halsey, 2019).

Although the negative association between host species diversity and parasite infection can be explained by alterations to the proportion of compatible hosts in a community, alternative mechanisms for the dilution effect have been proposed. In one model scenario, Keesing et al. (2006) described “encounter dilution” as a reduction in infection that occurs following the addition of a nonhost into a community The results of empirical studies have confirmed that the addition of species into a community, including those that are nonhosts or poor hosts, can have a strong influence on risk of infection into focal species (Rohr et al., 2015; Thieltges et al., 2008). Several mechanisms that lead to encounter dilution have been proposed, including a reduction in the numbers or performance of available infective stages by ingestion or attack by nonhosts, or by the loss of infective stages into nonhosts in which parasite development does not occur (Johnson & Thieltges, 2010). Encounter dilution can also occur when the presence of a nonhost changes the behavior of focal hosts and/or infective stages in a manner that reduces contact rates.

The difficulty in distinguishing among the proposed mechanisms that underlie the dilution effect limits our understanding of the biodiversity–disease relationship. In addition, the range in the types of host–pathogen systems that have been utilized to test for dilution effects is limited, further restricting our ability to make generalizations. Experimental tests for dilution effects in interactions involving fish as hosts, for example, are absent. Although results from field surveys provide preliminary support for the roles of both parasite dilution (Dargent et al., 2013) and encounter reduction (Gendron & Marcogliese, 2017), experimental tests are lacking. This key knowledge gap means that the effects of ongoing fish extirpations (Burkhead, 2012) and introductions (Vander Zanden, 1999) on parasite infection within contemporary host communities remain poorly known.

Fathead minnows (*Pimephales promelas*) that are infected with the brain‐encysting larvae (i.e., metacercariae) of the trematode, *Ornithodiplostomum ptychocheilus*, provide an ideal model to test for dilution effects and to distinguish among potential mechanisms. These small‐bodied cyprinids often dominate fish communities in the shallow, eutrophic wetlands that are characteristic of the North American Great Plains. In these types of wetland, fathead minnows are often the only fish present (Price et al., 1991). In other waterbodies in this region, they co‐occur with species of hypoxia‐tolerant fish, especially brook stickleback, *Culea inconstans*. In still other wetlands, fathead minnows have been introduced as a prey source to support local sportfish economies. In the latter case, fathead minnows tend to co‐occur with piscivores (e.g., pike, walleye, and rainbow trout) and a mix of other small‐bodied species of cyprinid fish (Nelson & Paetz, 1992). This range of fish community types provides an ideal scenario for tests of the host biodiversity–disease relationship. Furthermore, fathead minnows in these regions tend to be heavily infected with *O*. *ptychocheilus* larvae; prevalence is usually 100% and worm burdens in individual fish are often in the hundreds (Hendrickson, 1979; Sandland et al., 2001; Wisenden et al., 2012). Exposure occurs when free‐swimming larvae (= cercariae) that have been released from infected first intermediate hosts (the pond snail, *Physa gyrina*) penetrate the hosts’ epidermis, then migrate to the optic lobes and cerebellum via the peripheral and central nervous system (Matisz et al., 2010). The life cycle is completed when minnows with encysted larvae are ingested by fish‐eating birds, particularly great blue herons. The results from a series of studies involving the fathead–brainworm interaction have demonstrated that exposure of individual fish to known numbers of *O*. *ptychocheilus* cercariae is straightforward and that fish are easily maintained in both aquarium and mesocosms settings (e.g., Sacco et al., 2021).

In this study, we evaluate the host community structure–disease relationship by exposing fathead minnows to *O*. *ptychocheilus* cercariae within indoor aquaria and outdoor mesocosms that contained combinations of 1–4 species of small‐bodied fish: brook stickleback, long nose dace (*Rhinichthys cataractae*), and emerald shiner (*Notropis atherinoides*). In initial laboratory assays, we confirmed the nature of *O. ptychocheilus* specificity by exposing individuals of each species of fish to known numbers of cercariae and then counting the numbers of *O. ptychocheilus* cysts (i.e., brainworms). We then evaluated differences in mean brainworm counts between fish in monospecific tanks that contained only fathead minnows with counts in heterospecific tanks that contained combinations of the other species of fish. The range of host community types that spans the monospecific to heterospecific continuum provides an appropriate model to test the effects of host community diversity and host community identity on key components of parasite infection.

## 2 MATERIALS AND METHODS

### 2.1 Source of infective larvae

Naturally infected snails were used as a source of cercariae for experiments, following methods in Sacco et al. (2021). Adult *Physa gyrina* were collected from Coulee Creek Stormwater Pond, Lethbridge, Alberta (49°66’N, 112°78’W), between 5 July and 31 August 2018. Results from previous field surveys (Ahn, 2019) indicated that this site contained minnows and snails that were infected with *O*. *ptychocheilus* metacercariae and cercariae, respectively. A total of 294 adult snails was collected haphazardly from the perimeter of the pond and assessed for their infection status with larval trematodes. Infection status was assessed by placing individual snails in containers with clean water for 2 hr and observing for the release of free‐living cercariae. Of 159 snails collected on 5 July 2018, 19 individuals were infected with *O. ptychocheilus,* and of the 134 snails collected on 31 August, 12 individuals were infected. Infected snails were isolated and maintained under standard laboratory conditions with continual access to fresh Romaine lettuce and commercial fish food (Sandland & Goater, 2001).

### 2.2 Source of fish hosts

For the exposure experiments, juveniles (young‐of‐the‐year) of 4 fish species were collected in summer 2018 by seine net from several locations in southern Alberta. Fathead minnows were collected from a small pond located on the grounds of the University of Lethbridge, Alberta (49°68’N, 112°87’W). Emerald shiners and longnose dace were collected from the Oldman River along a stretch adjacent to the University (49°68’N, 112°86’W) and Brook stickleback were collected from McQuillan Reservoir (49°38'51.7"N 112°27'30.4"W). In southern Alberta, fathead minnows are a common and widespread species of cyprinid and are heavily infected with *O. ptychocheilus* metacercariae (Ahn, 2019; Sandland et al., 2001). The other species of fish tend to co‐occur with fathead minnows at most sites. Emerald shiners and longnose dace are more commonly found in moving water, while brook stickleback are more common in standing water. Even though all species of fish are sympatric at sites in southern Alberta, brook stickleback were syntopic at all sites.

All field‐collected fish were acclimated for at least 24 hr in laboratory aquaria at room temperature on a 16‐hr L: 8‐hr D light cycle. While under laboratory conditions, fish were fed ad libitum with a combination of commercial fish food (Tetramin®) and zooplankton (copepods and *Daphnia*) collected from a local reservoir. Aquaria water was changed every 3 days. For the mesocosm experiments, an additional transfer was performed that involved moving fish from laboratory aquaria to outdoor holding tanks. These fish were given an additional 24 hr of acclimation in outdoor holding tanks before transfer to the mesocosms.

A subset of 30 individuals of each species of fish was necropsied immediately after collection to determine their natural rates of exposure to *O. ptychocheilus*. Exposure to *O. ptychocheilus* cercariae starts in mid‐ to late July and peaks in late summer/early fall (Sandland et al., 2001). Thus, the presence of encysted *O. ptychocheilus* metacercariae in the juvenile fish resulted from exposure to cercariae during the previous late summer/fall.

### 2.3 Relative host susceptibility to *O. ptychocheilus* cercariae

Results from a limited number of host surveys have indicated that in ponds and lakes where fathead minnows co‐occur with other species of fish, including other “minnows” in the family Cyprinidae, *O. ptychocheilus* cercariae tend to be host‐specific (Sandland et al., 2001; Wisenden et al., 2012). However, experimental tests of host–parasite compatibility have not been completed. To determine the relative compatibility of hosts used in these experiments, 14 individuals of each species were exposed to *O. ptychocheilus* cercariae. Size‐matched samples of fathead minnow, emerald shiner, and longnose dace were selected haphazardly from the holding tanks and placed into 200‐mL plastic containers that held aquarium‐grade water. Brook stickleback were already known to be noncompatible with *O. ptychocheilus* cercariae prior to the experiment (Ahn, 2019; Sandland et al., 2001). Ten *O. ptychocheilus*‐infected snails were placed in 200 ml of water and placed under a light source for 3–4 hr. Once cercariae release was observed, three 1 ml aliquots of the cercariae‐filled water was collected and immersed in 70% ethanol (Sandland & Goater, 2000). The mean number of cercariae was calculated from counts made under a gridded Petri dish to obtain replicate densities of cercariae/200 ml (Sandland & Goater, 2000). The volume corresponding to 50 cercariae was then added to 200‐mL containers containing an individual fish for 2 hr. Cercariae‐exposed fish were then transferred into aquaria and housed according to species. Standard necropsy procedures (Sandland & Goater, 2000) were used to assess metacercariae intensity at 14 days post‐exposure. Complete maturation of *O. ptychocheilus* metacercariae requires a minimum of 8 weeks (Matisz et al., 2010). Thus, new infections resulting from the experimental exposures were easily distinguishable from prior exposures. At necropsy, the wet weight (0.1 g) and standard length (mm) of each host were assessed.

### 2.4 Laboratory experiment

For the aquaria experiment, fish were transferred to 3‐L experimental plastic containers (H: 17 cm × L: 21cm × W: 14cm) from the holding tanks and provided 24‐hr acclimation prior to exposure to cercariae. This experiment was completed in the Aquatic Research Facility at the University of Lethbridge. The experiment was set up with a randomized block design with 5 replicates of each of the following 7 treatments: 2 fathead minnows; 4 fathead minnows; 8 fathead minnows; 2 fathead minnows +2 emerald shiners; 2 fathead minnows +2 longnose dace; 2 fathead minnows +2 brook sticklebacks; 2 fathead minnows +2 emerald shiners +2 longnose dace +2 brook sticklebacks. Following the 24‐hr acclimation period, fish in each container were exposed to *O. ptychocheilus* cercariae using methods similar to those described for the relative compatibility experiment. In this experiment, fish in a container were batch‐exposed to 200 cercariae from 10 *O*. *ptychocheilus*‐infected snails on 8 September 2018. The survivors in a container were necropsied 10 days post‐exposure to obtain metacercariae counts. Fish were fed zooplankton (*Daphnia* and copepods) daily, and water was changed every 2 days. Live food was used to prevent fouling of the water in the containers and because all species of fish readily consumed these prey items (some species did not consume Tetramin^®^). Food quantity/container was determined on a per capita basis by the density of fish in the containers and was estimated as approximately 1 ml of concentrated zooplankton per individual/day.

### 2.5 Mesocosm experiment

Sixteen 1,200 L outdoor mesocosms and 4 outdoor holding tanks were set up within a fenced area on the University of Lethbridge campus. The methods used to set up the mesocosms and to batch‐expose fathead minnows to *O. ptychocheilus* cercariae followed our standard protocol (Stumbo et al., 2012). In brief, mesocosms were filled with irrigation water and dried *Typha sp*. reeds (800 g) from a local wetland on 18 May 2018. A 500 ml inoculation of concentrated phyto‐ and zooplankton was collected from 4 local ponds and added to each mesocosm on 15 June and 12 July 2018. Each mesocosm was covered with a mesh lid during the duration of the experiment to prevent the colonization by predatory invertebrates. The mesocosms were left undisturbed until 27 July 2018 to allow for the reproduction and development of plankton that would provide a food source for the experimental populations of fish.

Experimental fish were transferred from outdoor holding tanks to the mesocosms on 27 July 2018 and were acclimated for a minimum of 24 hr prior to exposure to *O. ptychocheilus* cercariae. The design included 4 replicates of 4 treatments: 30 fathead minnow, 60 fathead minnow, 30 fathead minnow +30 emerald shiner, 30 fathead minnow +30 longnose dace. Cercarial exposures started on 30 July 2018 with cercariae from 10 *O*. *ptychocheilus*‐infected *P. gyrina*. A total of 10,000 cercariae was immersed into each mesocosm between 1:00 and 5:00 p.m. each day for 7 consecutive days. Fish necropsies were conducted starting 16 August 2018, at least 10 days following the last day of cercariae exposure.

### 2.6 Analyses

The compatibility of each fish species to *O. ptychocheilus* cercariae was determined as “mean percent encystment,” calculated as the numbers of larvae encysted in the brain (i.e., brainworm intensity) relative to the numbers of cercariae used in exposures. A Kruskal–Wallis analysis of variance was used to test difference in compatibility between host fish species (any species that had >0 mean percent encystment of brainworm) following a square‐root transformation to meet the assumptions of the test.

The analyses used to test for the effects of density were limited to the monospecific fathead minnow treatments. By extension, to test for the effects of host diversity on brainworm intensity, the effect of diversity was only tested between treatments with the same total fish density. A Bonferroni correction for multiple comparisons was applied to our p‐value for interpretations of the results.

To test the effects of density (*n* = 2, 4, 8) for the laboratory experiments, a linear mixed‐effects (LMM) model with brainworm intensity in fathead minnows as the dependent variable, host density as a fixed effect, host body condition (the residual of a regression between wet weight and standard length) as a covariate, and experimental container as a random effect. Paralleling the model used to test the effects of density, LMMs were used to test for the effect of community composition on brainworm intensity in fathead minnows (one model for community densities of 4 fish, and another model for community densities of 8 fish) with community composition as a fixed effect, host body condition as a covariate, and experimental container as a random effect.

Analyses for density and composition effects for the mesocosm experiments were conducted similarly. Density effects (*n* = 30, 60) were tested with a generalized linear mixed‐effects model (GLMM) with a negative binomial error distribution. Host density was a fixed effect, host body condition was a covariate, and experimental container was a random effect. To test for the effects of community composition (60 fathead minnow, 30 fathead minnow +30 emerald shiner, 30 fathead minnow +30 longnose dace) in the mesocosm experiment, a GLMM with a negative binomial error distribution was constructed with community composition as a fixed effect, host body condition as a covariate, and experimental container as a random effect. All statistical analyses were performed using R (version 3.4.1) and the models were performed using the lme4 package.

## 3 RESULTS

### 3.1 General patterns of metacercariae infection in fish

The prevalence and mean abundance of brainworm in field‐collected juvenile fish varied by host species (Table 1). Longnose dace were uninfected, whereas 95% of the fathead minnows and emerald shiners were infected with less than 2 larvae.

**TABLE 1:** Infection characteristics of *Ornithodiplostomum ptychocheilus* larvae (metacercariae) in three species of fish collected from sites in Southern Alberta (*n* = 30 juveniles/site) and their relative susceptibility to *O. ptychocheilus* following exposure to 50 infective larvae

Host	Date of collection	Mean (±*SD*) abundance	Prevalence (%)	Mean (±*SD*) host length	Mean (±*SD*) percent recovery^a^
Fathead minnow	11–25 July	0.7 (± 0.2)	36.7	28.3 (± 2.2)	56 (± 21)
Emerald shiner	24 July–7 September	1.0 (± 0.3)	46.7	48.4 (±5.1)	1 (± 0.03)
Longnose dace	14–25 July	0	0	34.8 (± 5.9)	0

^a^ Numbers of metacercariae in the brain divided by exposure dose × 100.

The results of the laboratory exposures showed that longnose dace were incompatible with *O*. *ptychocheilus* cercariae. Fathead minnows were susceptible to cercariae with a mean encystment of 56%, whereas encystment of brainworms in emerald shiners was 1% (Table 1). The difference in brainworm encystment between fathead minnows and emerald shiners was significant (Kruskal–Wallis analysis of variance, $\chi_{1}^{2}$ = 21.606, *p* < .0001).

### 3.2 Laboratory experiment

**FIGURE 1:**
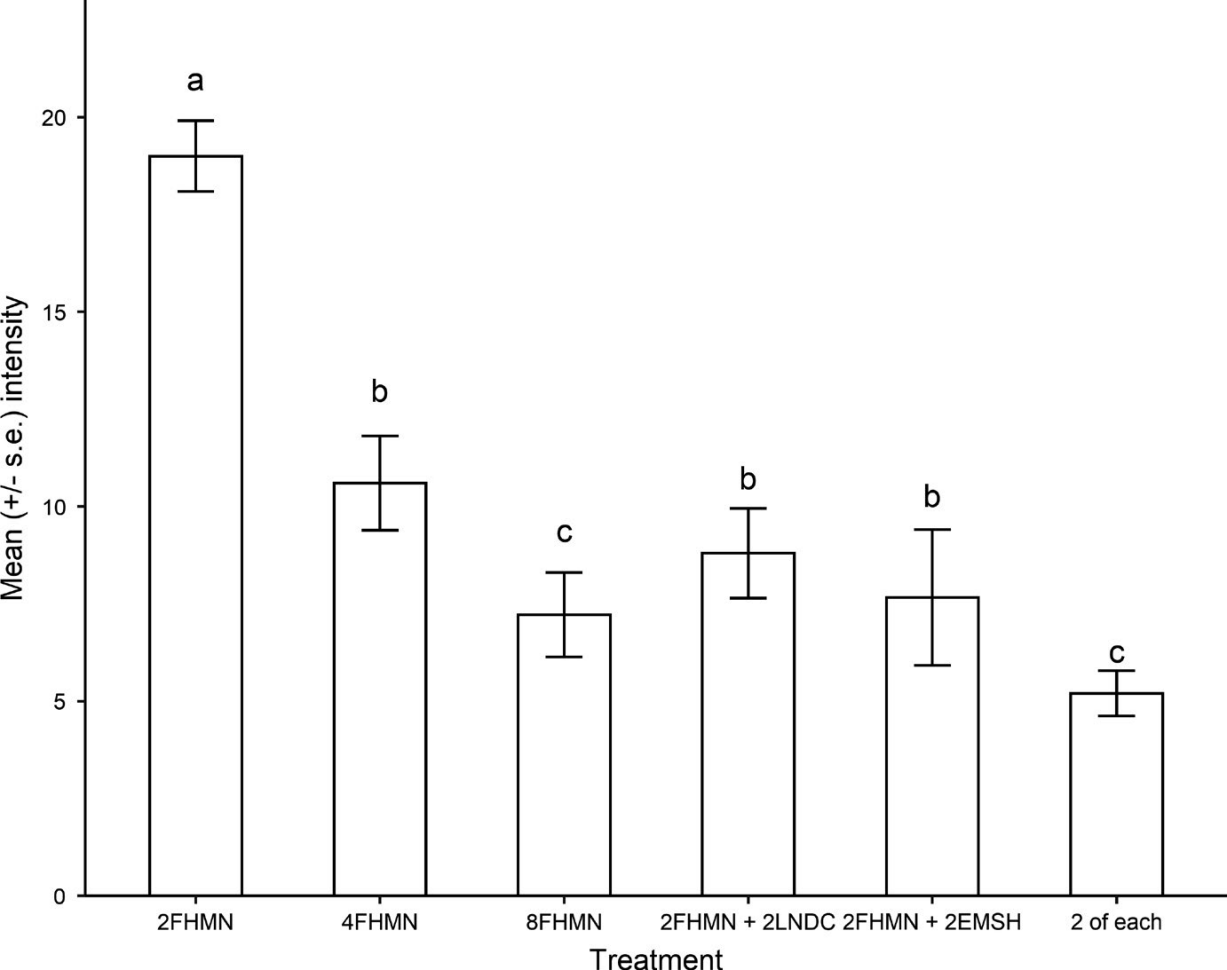
Effect of host density (total *n* = 2, 4, or 8 per container) and host diversity (FHMN = fathead minnow; EMSH = emerald shiner; LNDC = longnose dace; BKST = brook stickleback; 2 of each = 2 FHMN + 2 EMSH + 2 LNDC + 2 BKST) on the mean numbers of trematode larvae (metacercariae) in fathead minnows following exposure in indoor aquaria. Annotations indicate statistical significance (*p* < .05) for pairwise comparisons between heterospecific combinations and monospecific combinations involving fathead minnows at the same total fish density (i.e., 4FHMN treatment versus 2FHMN + 2LNDC)

There was a 44.2% decrease in mean brainworm intensity in individual fathead minnows between the 2‐ and 4‐density containers and a 31.9% decrease between individual minnows in the 4‐ and 8‐density containers (Figure 1; LMM, density: −0–1.82 ± 0.43, *df* = 11, *p* = .0013). There was a 27.4% decrease in mean brainworm intensity between minnows in the monospecific minnow tanks and minnows in the heterospecific tanks that contained emerald shiners; this decrease was close to significant (Figure 1; LMM, treatment: −4.17 ± 2.2, *df* = 10, *p* = .09). Mean brainworm intensities in the minnow tanks were not significantly different from those in the heterospecific tanks with longnose dace (*n* = 10) (Figure 1; LMM, treatment: −0.64 ± 2.01, *df* = 10, *p* = .76). There was also no significant difference in mean brainworm intensity in minnows in the monospecific 8 minnow‐only treatment (*n* = 18) compared to minnows in the mixed treatment (*n* = 5) that contained shiners, dace, and sticklebacks (Figure 1; LMM, treatment: 1.73 ± 2.42, *df* = 6, *p* = .50). Whereas relatively low rates of minnow mortality occurred in all containers during the experiment, there was unusually high mortality in the 2 fathead minnow +2 brook stickleback containers. This treatment was not included in analyses.

### 3.3 Mesocosm experiment

**FIGURE 2:**
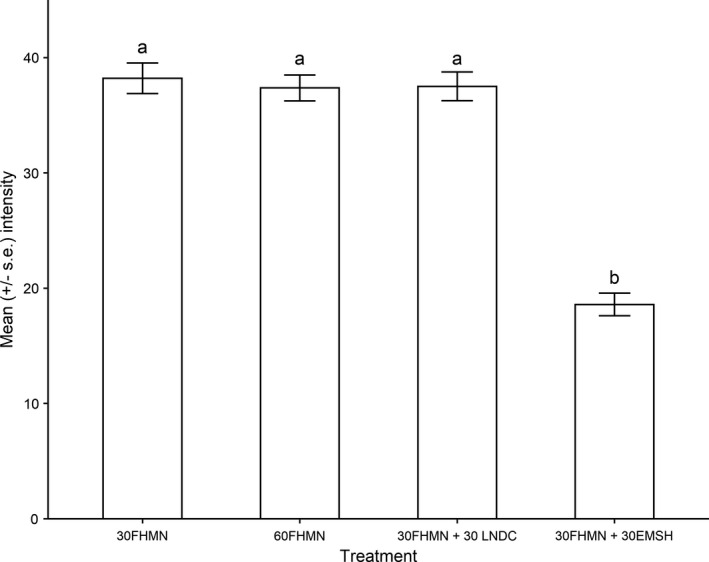
Effect of host density (total *n* = 30 or 60 per container) and host diversity (FHMN = fathead minnow; EMSH = emerald shiner; LNDC = longnose dace) on the mean numbers of trematode larvae (metacercariae) in fathead minnows following exposure in outdoor mesocosms. Annotations indicate statistical significance (*p* < .05) for pairwise comparisons between heterospecific combinations and monospecific combinations involving fathead minnows at the same total fish density (i.e., 60FHMN treatment versus 30FHMN + 30LNDC treatment)

There was no significant difference in brainworm intensity between minnows from the low‐ (*n* = 78) and high‐density (*n* = 134) mesocosms (Figure 2; GLMM, density: −0. 022 ± 0.16, *df* = 207, *p* = .84). Furthermore, mean brainworm intensities in the fathead minnows from heterospecific mesocosms with dace were not significantly different from those in their monospecific mesocosms at the same density (Figure 2; GLMM, treatment: 0.043 ± 0.13, *p* = .74). In contrast, mean brainworm intensity in fathead minnows was 50.9% less in the heterospecific mesocosms that contained emerald shiners compared to the monospecific mesocosms that contained minnows only (Figure 2). The difference in mean brainworm intensity in the heterospecific versus monospecific mesocosms was significant (GLMM, treatment: −0.73 ± 0.13, *p* < .0001).

## 4 DISCUSSION

Our results show that the structure of a host community has a strong effect on parasite infection rate, but only under a restrictive set of conditions. *Ornithodiplostomum ptychocheilus* infection in fathead minnows was markedly reduced when they co‐occurred in tanks with emerald shiners compared to when they were by themselves or when they co‐occurred with two other species of nonhosts. Infection reduction within minnow + shiner combinations was highest in the mesocosms and also occurred, but to a lesser extent, in laboratory aquaria. Thus, when host biodiversity increased from one to two species of host through the addition of emerald shiners, there was a marked reduction in infection. These results are consistent with the results of other empirical tests in which increased host species richness resulted in decreased rates of parasite infection (reviewed in Civitello et al., 2015). However, in all other pairwise host community combinations, infection was equivalent to those in the monospecific minnow treatments that contained the same total numbers of fish. Furthermore, infection was not significantly affected when exposure occurred within laboratory containers that held mixtures of 4 host species, including emerald shiners. These results indicate that infection reduction only occurred under specific conditions that involved co‐occurrence with a single species of host. These results also emphasize the context‐dependent nature of the disease–biodiversity relationship and the importance of host species identity as a key mechanism affecting rates of parasite infection within complex host communities (Halliday & Rohr, 2019; Johnson et al., 2019; Wojdak et al., 2014).

The results from the laboratory experiment showed that the reduction in infection that occurred when minnows co‐occurred with nonhost dace and nonhost stickleback could not be distinguished from the reduction that occurred when additional minnows were added to containers. When there was a total of 4 fish in a container, infection in the two minnows was the same regardless of the mix of co‐occurring species present. Thus, neither of these nonhosts had a significant influence on the availability of cercariae in the water column for penetration into minnows. Results of our laboratory infection trials show that among the hosts we tested, *O. ptychocheilus* cercariae are host‐specific. Cercariae could penetrate and establish in minnows, did so rarely in shiners, but not in dace or stickleback. These empirical results are consistent with infection data from our field surveys and from previous surveys involving fathead minnows collected from other locations in Alberta (Ahn, 2019; Sandland et al., 2001). One general implication is that for host‐specific parasites, changes in biodiversity through local host extirpations or host introductions may, under some conditions, be inconsequential to rates of infection into focal hosts.

In contrast, mean brainworm intensity in minnows within minnow + shiner containers was reduced by up to 50% compared to intensities in minnows from minnow +dace and minnow + stickleback containers kept at the same total fish density. This result can be explained by a reduction in the numbers of cercariae available for penetration of minnows following their loss within shiners. In this scenario, shiners would act as a sink for cercariae that attempt to penetrate a nonhost are either killed during the attempt, or die during the process of migration and encystment within the host. This form of dilution has been demonstrated in other studies involving aquatic trematodes that have free‐living larval stages (Johnson et al., 2008; Laracuente et al., 1979). We did not assess the fate of cercariae in exposed fish, nor did we directly assess if cercariae attempt to penetrate these nonhosts. The results of previous studies in our laboratory show that the penetration and migration of *O. ptychocheilus* cercaria is a complex process, ultimately leading to obligate site selection within specific regions of the optic lobes in fathead minnows (Matisz et al., 2010). For this species and for other tissue‐specific trematodes (e.g., the “eyeflukes” of freshwater fish), tight host specificity appears to be common (Locke et al., 2015) and cercariae have not been documented to recognize, or penetrate, noncompatible hosts. Although we cannot discount the possibility that cercariae were lost following their penetration of shiners (but not sticklebacks or dace), we consider this to be an unlikely explanation for infection reduction in the minnow + shiner combinations.

Alternatively, the observed reduction in brainworm intensity could be caused by a decline in the rates that minnows encountered cercariae within minnow + shiner containers. For cercariae–fish interactions, host behaviors associated with activity, shoaling, and reproduction are known to influence rates of infection into individual hosts (Wisenden et al., 2009). Our anecdotal observations of individual fish within the mesocosms indicated that minnows tended to form mixed shoals with emerald shiners but not with dace or stickleback. Interspecific shoaling involving cyprinids is associated with changes in the swimming behavior of individual fish (Tang et al., 2017), their positioning within a shoal (Allan, 1986), and with overall shoal architecture (Pollock et al., 2006). Results from our previous work have indicated that rates of infection of *O. ptychocheilus* cercariae into minnows are influenced by minnow behaviors such as host activity (Shirakashi & Goater, 2005) and central positioning within a conspecific shoal (Stumbo et al., 2012). Therefore, interspecific interactions between shiners and minnows may cause alterations to shoal architecture that influence encounter rates with cercariae. The influence of shiners on other minnow behaviors such as microhabitat preference, place/time foraging, or overall activity may similarly influence encounter rates with cercariae. An intriguing and testable possibility for follow‐up studies is that changes in host biodiversity, perhaps via host introductions, may affect social conditions for competent hosts that ultimately influence their risk of exposure to parasites (Keesing et al., 2006).

Encounter dilution within minnow + shiner containers could occur via predation on cercariae by emerald shiners. The ingestion of cercariae (cercariophagy) by planktivorous fish has been documented in other fish–trematode systems (Kaplan et al., 2009). Similarly, indirect effects of cercariophagy would be important if shiners favored zooplankton prey that were present in the mesocosms that in turn preyed on cercariae. High rates of cercariaphagy by zooplankton, especially by copepods, have been documented (Mironova et al., 2019). Indeed, one explanation for the observed differences in rates of infection between the mesocosm and laboratory studies may lie in the contrasting opportunities for cercariaphagy in the two settings. Tracer studies designed to evaluate the relative rates of cercarial ingestion by host and nonhost species would be a fruitful direction to better understand the role of ingestion by nonhosts in encounter dilution.

Lastly, encounter dilution within minnow + shiner containers could occur if shiners influenced the swimming performance and infectivity of cercariae in the mixed containers. Trematode cercariae are millimeter‐scale, short‐lived (ca. 12–24 hr), nonfeeding, and free‐swimming (Goater et al., 2014). Following their daily release from snails, they engage in efficient, but temporary, swimming behaviors that are driven by the muscular action of a posterior tail (Combes et al., 1994). For many species of trematode, these complex larval behaviors lead to the aggregation of cercariae at the air–water interface. Since emerald shiners tend to prefer swimming near the surface of the water column (Trautman, 1981), it is possible that their activity influenced the behavior of individual cercariae in a manner that influenced their rate of encounter with minnows. As indicated previously for shiner‐induced impacts on host behavior, the contrasting results observed between the laboratory and mesocosm studies may result from shiner‐induced impacts on cercariae behavior that influence encounter rates with fathead minnows.

Our results that emphasize the role of host identity in the dilution effect have important implications for our understanding of biodiversity–disease relationships. Our results are particularly relevant to our understanding of the effects of exotic fish introductions into native communities that are dominated by host–specialist parasites. Poulin (1992) showed that approximately 50% of the trematodes reported from Canadian freshwater fishes were restricted to a single host species. Thus, when an exotic species of fish is introduced outside its native range, it is highly likely to be exposed to larval stages of host‐specific parasites. Our results imply that the consequence of such an introduction on rates of infection can be expected to be highly species‐dependent. On the one hand, we might expect a nonhost introduction to have little to no effects on infection, a scenario parallel to the addition of sticklebacks and dace into minnow containers in the experiments reported here. On the other, we might expect modified rates of infection in cases where introduced nonhosts alter key host or parasite behaviors that influence host–parasite encounter rates—a scenario parallel to our addition of shiners in containers with minnows. We contend that in light of the pace of anthropogenic change occurring in contemporary fish communities (Tickner et al., 2020), there is increasing urgency to characterize and understand this variation in outcome.

## CONFLICT OF INTEREST

The authors have no conflicting interests.

## AUTHOR CONTRIBUTION

**Sangwook Ahn:** Conceptualization (equal); Data curation (lead); Formal analysis (lead); Funding acquisition (supporting); Investigation (lead); Methodology (lead); Project administration (lead); Visualization (equal); Writing‐original draft (equal); Writing‐review & editing (equal). **Cameron Goater:** Conceptualization (equal); Data curation (equal); Formal analysis (supporting); Funding acquisition (lead); Investigation (supporting); Methodology (equal); Project administration (supporting); Supervision (lead); Visualization (equal); Writing‐original draft (equal); Writing‐review & editing (equal).

## PERMITS

Animal handling was approved by the University of Lethbridge Animal Welfare Committee (Protocol 1,418), and animal collections were approved by Alberta Tourism, Parks and Recreation Research and Collection (Permit #: 18‐536).

## Data Availability

Under review in Dryad: https://doi.org/10.5061/dryad.0p2ngf21r
